# Fungal literature records database of the sub-Antarctic Region of Aysén, Chile

**DOI:** 10.3897/BDJ.9.e75951

**Published:** 2021-12-29

**Authors:** Laura Sánchez-Jardón, Laura del Rio-Hortega, Noemi Núñez Cea, Mario Mingarro, Paloma Manubens, Sebastián Zambrano, Belén Acosta Gallo

**Affiliations:** 1 Universidad de Magallanes, Coyhaique, Chile Universidad de Magallanes Coyhaique Chile; 2 RIAMA: Red de Investigadores Actuando por el Medio Ambiente, Madrid, Spain RIAMA: Red de Investigadores Actuando por el Medio Ambiente Madrid Spain; 3 Universidad Complutense, Madrid, Spain Universidad Complutense Madrid Spain; 4 Museo Nacional de Ciencias Naturales-CSIC, Madrid, Spain Museo Nacional de Ciencias Naturales-CSIC Madrid Spain

**Keywords:** Chilean Patagonia, macrofungi, lichens, Mycobiota, SiB-Aysén, GBIF

## Abstract

**Background:**

To this day, merely 8% of all estimated fungi species are documented and, in certain regions, its biodiversity is practically unknown. Inside the Fungi Kingdom, macrofungi and lichens assume a critical part in the ecosystem functionality and have a historical connection to mankind's social, clinical and nutritious uses. Despite their importance, the diversity of these groups has been widely overlooked in the sub-Antarctic Region of Chile, a crucial area in the study of climate change due to its extraordinary biodiversity and its proximity to Antarctica. Few studies regarding both groups have been conducted in this sub-Antarctic Region and the data are still scarce and inaccessible, as these are only published in specialised journals, unreachable to local communities.

**New information:**

This publication presents a records compilation available in previous published scientific and technical reports on macrofungi and lichen diversity. In total, 1263 occurrence records of 618 species (341 records of 251 macrofungi species and 922 records of 367 lichen species) were digitised and integrated into the regional platform Biodiversity Information System for Aysén (SIB-Aysén) and into GBIF. Here, we provide the fullest dataset on one of the most diverse group of living beings in one of the the least-known world regions.

## Introduction

The biodiversity of the Fungi Kingdom is immense, including organisms with varied forms, colours and life habits, from macroscopic to microscopic sizes, being present in all ecosystems on Earth, both terrestrial and marine (Tedersoo et al. 2014). They are essential for ecosystem functionality (Öpik et al. 2006), due to their participation in the organic matter decomposition processes and the symbiotic relationships with other living beings (Dighton and White 2017). They also provide unique uses as food, medicinal or cultural with great potential for industrial development (Furci 2013, Hyde et al. 2019). Macrofungi and lichens are classified within this kingdom, in addition to yeasts and moulds. Macrofungi are non-photosynthetic heterotrophic free-living organisms with macroscopic fruiting bodies which may grow on various substrates (Dighton and White 2017). According to their nutrition, they can be classified as: saprophytes, which obtain nutrients and energy from decaying plant material; parasites, which derive nutrients from their host from other co-inhabitants; and symbiotics, which associate with other organisms with a mutual benefit to each other (Lazo 2001, Furci 2008). Lichens, on the other hand, are symbiotic organisms in which a fungi, known as mycobiont, is in a close symbiosis with microalgae or a cyanobacterium, known as photobiont (Hawksworth and Grube 2020). As photosynthetic organisms and pioneers of ecosystems (Vargas-Castillo and Morano 2014, Bjerke et al. 2003), macrofungi and lichens have essential ecological functions as they participate in the carbon and nitrogen cycles, act as primary colonisers in succession and serve as bioindicators (Furci 2013). They are found in all types of habitats and substrates, colonising rocks, logs, the ground and other organisms (Quilhot et al. 2012). Taxonomically, most lichens and macrofungi are classified in the Divisions Basidiomycota and Ascomycota, which differ in the way they produce and discharge spores (Lazo 2001).

It is estimated that there are between 2.2 to 3.8 million fungi species in the world, but even though methodological approaches are rapidly improving the available knowledge, less than 8% are currently known (Blackwell 2011, Hawksworth and Lücking 2017, Tedersoo et al. 2014). In Chile, one of the least known biodiversity groups is the Fungi Kingdom, with only 1500 known species (Furci 2008) and specifically, in the Aysén Region, the lack of knowledge is even greater. This Region, belonging to the sub-Antarctic zone of Chile together with the Magallanes Region, is one of the most pristine places on the planet, being crucial in the study of climate change due to its extraordinary biodiversity and its proximity to Antarctica (Osorio et al. 2002, Rozzi et al. 2020). This Region represents the biogeographic limits of the extreme Antarctic conditions (Macaya and Zuccarello 2010), making it an extremely fragile area, but with an extraordinary natural heritage since it houses a great variety of terrestrial and aquatic ecosystems (Álvarez et al. 2010). Due to the harsh conditions that occur in this territory composed of mountain ranges, deserts and glaciers, the fungi biodiversity is detrimentally affected, with only a few species being able to settle (Furci 2008). These hostile intrinsic conditions have even limited the fungi biodiversity research in this area in the past, but fortunately, there are increasing initiatives that contribute to the local fungi biodiversity knowledge directly from the government (Corporación Nacional Forestal 2009, Corporación Nacional Forestal 2010, Corporación Nacional Forestal 2012), as well as a regional platform that has been developed under the same standards as Darwin Core (DwC), the Biodiversity Information System for Aysén or SiB-Aysén (https://kataix.umag.cl/sib-aysen/). SiB-Aysén fosters citizen participation as it is an interactive platform that allows users to enter their records according to a validation protocol (Sánchez-Jardón et al. 2021).

One of the missions of this initiative involves the gathering of previous studies data, incorporating them into biodiversity information systems and making them accessible for consultation (Chavan and Penev 2011). This data integration effort is especially relevant in the remote areas of the planet, such as the sub-Antarctic Region of Chile, where sampling tasks are extraordinarily expensive and, therefore, more scarce. As long as the information remains disseminated in multiple sources, it is easier for the disparity to occur in subsequent sampling efforts (Kühl et al. 2020), causing over-representation of records in some areas, generally those closer to human populations (Barbosa et al. 2013). In any case, behind each GBIF dataset, there is considerable digitisation, compilation and validation efforts. Systematising the sampling or collection data is common, but adapting the databases to certain standards, such as Darwin Core, so that they are available to the public, represents an additional effort (Heberling et al. 2021).

The purpose of this work is to collect the macrofungi and lichen data available in other published scientific and technical reports to incorporate it in the Biodiversity Information System for Aysén (SIB-Aysén) and the GBIF. This way, the biodiversity information can be consulted and used in management and conservation programmes, as well as complemented by other scientific and citizen observations in future investigation and citizens science projects (Penev et al. 2017, Rozzi et al. 2020).

## Project description

### Title

Fungal literature records database of the sub-Antarctic Region of Aysén, Chile (https://kataix.umag.cl/sib-aysen/)

### Personnel

Laura Sánchez-Jardón

## Sampling methods

### Study extent

The geographic extent (48°42'55"S to 44°8'3"S; 74°25'32"W to 71°32'18"W) of the digitised dataset corresponds to the Region of Aysén del General Carlos Ibáñez del Campo (XI Region) with an area 109.052 km^2^, representing about 14% of the Chilean territory. This Region is situated in the sub-Antarctic zone of Chile, which comprises both the Magallanes and Aysén administrative regions, both having an extraordinary biodiversity, representing one of the most pristine areas on the planet, unique for its proximity to Antarctica and with significant implications in the study of climate change (Osorio et al. 2002, Rozzi et al. 2020).

### Sampling description

The digitisation aims to summarise the fungi species occurrences accumulated in previous mycological studies and published in reviewed scientific literature (Filippova et al. 2020). To fulfil the aim of this work, an exhaustive review of the scientific literature, related to the diversity of macrofungi and lichens in the Aysén Region, Chile, was carried out using scientific content search engines, such as Web of Science and Google Scholar. A total of 16 references were found (nine specialised journals, four books and three technical documents) that contained records or suggested the presence of macrofungi and lichen species within the territory of the Aysén Region (Bjerke et al. 2003, Lazo 2001, Osorio et al. 2002, Quilhot et al. 2002a, Quilhot et al. 2002b, Quilhot et al. 2002c, Furci 2008, Corporación Nacional Forestal 2009, Corporación Nacional Forestal 2010, Furci 2013, Corporación Nacional Forestal 2012, Quilhot et al. 2012,Ramírez et al. 2014,Sandoval-Leiva 2014, Vargas-Castillo and Morano 2014, Furci 2018). This data collection started with a project FIC from the Aysén Regional Government known as “Biodiversity Information System for Aysén, SIB-Aysén (BIP 40000522-0) and continued with the project “Hongusto: social innovation regarding wild and cultivated edible mushrooms from the Aysén region (CORFO 15IS-46635)”. Eventually, it will be complemented by the citizens’ observations associated with the SIB-Aysén. Eventually, the citizens’ observations associated with the SIB-Aysén will complement this study.

### Quality control

The taxonomic nomenclature used has been updated according to MycoBank (available at https://www.mycobank.org/). The geographic coordinates have been systematised from what was reported by the authors in the publications; when only the locality was mentioned, it has been approximated using Google Earth; in both cases, the coordinates have been validated using the GBIF tool.

### Step description

**Figure 1.**
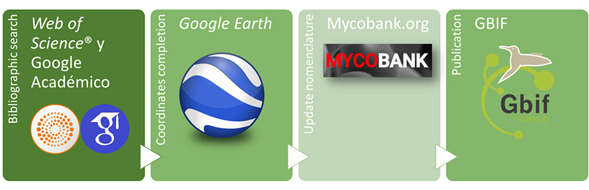
Flowchart depicting major steps in dataset development and publishing.

The records were systematised in a database with taxonomic, geographic and temporal information. The coordinates indicated by the authors were assumed; when not indicated in the publication, they were obtained from the locality names using GoogleEarth®. The substrate information of the lichens, in case that it was provided by the authors, was classified as follows according to the reviewed literature: epiphytes when only growing on trees or shrubs; epiphytic-lignicolous when growing on rotten wood or decaying organic matter; folicolous when growing on living tree leaves; muscicolous when growing on mosses; saxicolous when growing on rocks and terricolous when growing on the ground. The taxonomic nomenclature used in each publication was updated according to MycoBank. Ultimately, this information was systematised according to the DwC standard (Fig. 1) and uploaded to GBIF (Sánchez-Jardón et al. 2021). The complete dataset is available in Darwin Core Archive format via the Global Biodiversity Information Facility (GBIF).

## Geographic coverage

### Description

**Figure 2.**
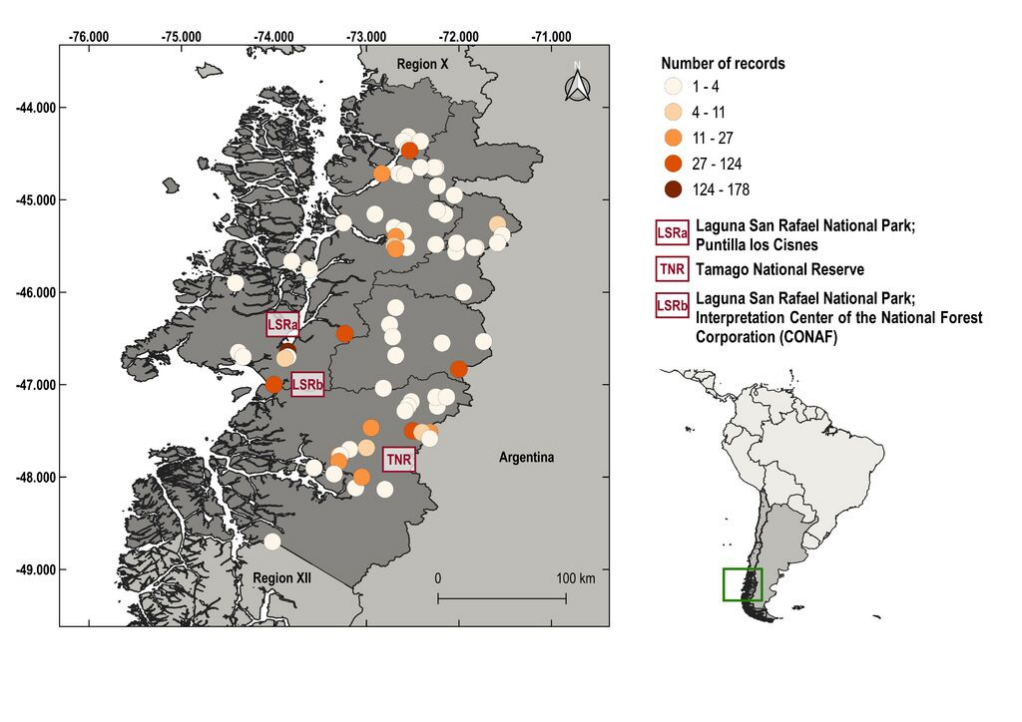
Number of Fungi records in Aysén, Chile. Localities with the highest numbers are indicated.

The records were distributed throughout the entire Aysén Region, with a slightly greater presence in the south-eastern half of the Region (Fig. 2). Broadly, the density of records is low (from 1 - 4 records per locality), although there are areas with a higher density (up to 178 records in the same locality). The locations with the highest numbers of records are Puntilla de los Cisnes, in the National Park Laguna de San Rafael (178 records), Tamago National Reserve (123 records) and Interpretation Center of the National Forest Corporation (CONAF) in Laguna de San Rafael too (76 records).

### Coordinates

44° 8' 3"S and 48° 42' 55"S Latitude; 74° 25' 32"W and 71° 32' 18"W Longitude.

## Taxonomic coverage

### Description

In total, 1263 records have been collected from 618 taxa belonging to the Fungi Kingdom, namely macrofungi (341 records of 251 species) and lichens (922 records of 367 species). As shown in Table 1, the order of macrofungi with the most abundance of records is Agaricales (203 records of 158 species), which accounts for 60% of the macrofungi records, followed by Pezizales (27 records of 18 species); Boletales (19 records of 11 species); Polyporales (16 records of 10 species); Cyttariales (12 records of 6 species); Russulales (12 records of 8 species); Geastrales, Helotiales and Gomphales (7 records of 4, 7 and 5 species, respectively); Dacrymycetales (6 records of 3 species); Hymenochaetales (5 records of 4 species); Geoglossales and Xylariales (4 records of 3 species each); Phallales and Tremellales (3 records of 2 and 3 species, respectively); Pucciniales and Hysterangiales (2 records of 1 and 2 species, respectively); and finally, Auriculariales and Thelephorales (1 records of 1 species each). Regarding lichens, the orders with the most abundance of records are Lecanorales (437 records of 165 species) and Peltigerales (375 records of 142 species), both accounting for around 88% of the records, followed by Pertusariales (20 records of 9 species); Baeomycetales and Teloschistales (14 records of 10 and 8 species); Rhizocarpales (13 records of 5 species); Caliciales (12 records of 8 species); Umbilicariales (10 records 6 species); Verrucariales (7 records of 4 species); Agaricales (6 records of 2 species); Gyalectales (5 records of 1 species); Arthoniales (4 records of 3 species); Graphidales (3 records of 2 species); and finally, Candelariales and Ostropales (1 record of 1 species each). A complete list of species and number of records is available in Suppl. materials 1, 2.

At family level, amongst the macrofungi, the most diverse family is Strophariaceae with 30 records of 27 species (Table 2), followed by Cortinaceae and Agaricaceae with 28 records of 21 and 22 species, respectively. The macrofungi species with the most records are *Cyttariadarwinii* and *Cortinariusmagellanicus* (4 records each), followed by *Ganoderma australe, Gyromitra antarctica, Morchella conica, Ramaria flava* and *Trametesversicolor* amongst 20 other species with 3 records each. In the case of lichens, the most frequent family is Parmeliaceae with 277 records of 85 species (Table 2), followed by Peltigeraceae (251 records of 88 species) and Pannariaceae (83 records of 34 species). The species with the most records are *Menegazziaglobulifera* (13 records) and *Leifidiumtenerum* (12 records), *Pseudocyphellariacrocata* and *Chloreamalacea* (11 records each). The number of records per species is available in Suppl. materials 1, 2.

## Traits coverage

**Figure 3.**
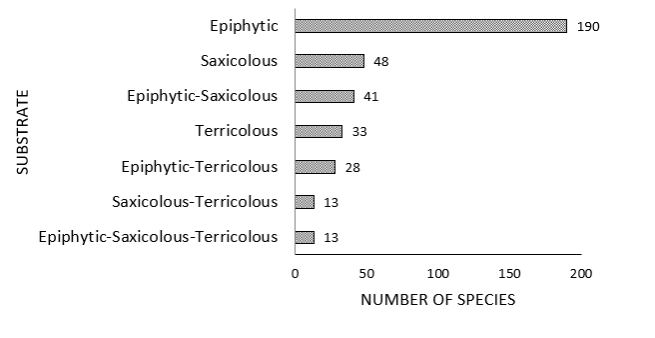
Number of lichen species according to their substrate.

Considering the type of substrate in which the lichen grow, most of the recorded lichen species are epiphytic (which includes epiphytic, lignicolous, folicolous and muscicolous of the database habitat categories) with 192 of 367 species with substrate information (Fig. 3), followed by 48 saxicolous species; 41 epiphytic-saxicolous species and 33 terricolous species. The remaining species were epiphytic-terricolous (28 species); saxicolous-terricolous (13 species) and epiphytic-saxicolous-terricolous (13 species). Regarding temporary coverage, the records cover a period of 51 years, from 1967 to 2018.

## Temporal coverage

**Data range:** 1984-11-01 – 2014-1-01.

## Usage licence

### Usage licence

Creative Commons Public Domain Waiver (CC-Zero)

## Data resources

### Data package title

Fungal literature records database of the sub-Antarctic Region of Aysén, Chile

### Resource link

https://www.gbif.org/es/dataset/8fbf6bab-3c5f-4397-8eac-362a4d582b3c

### Alternative identifiers

https://doi.org/10.15468/fuwe8e; http://gbif-chile.mma.gob.cl/ipt/resource?r=fungi_sib_aysen

### Number of data sets

1

### Data set 1.

#### Data set name

Diversidad potencial del Reino Fungi (macrohongos y líquenes) en el Sistema de Información en Biodiverisad para Aysén (SIB-Aysén), Chile

#### Data format

Darwin Core

#### Number of columns

53

#### Description

The dataset includes a table in Darwin Core format with 53 fields and 1263 records.

**Data set 1.** 

Column label	Column description
type	The nature or genre of the resource. The name of the class that defines the root of the record.
language	The language of the resource.
license	A legal document giving official permission to do something with the resource.
rightsHolder	A person or organisation owning or managing rights over the resource.
accessRights	Information about who can access the resource or an indication of its security status.
InstitutionID	An identifier for the institution having custody of the object(s) or information referred to in the record.
collectionID	An identifier for the collection or dataset from which the record was derived.
institutionCode	The name (or acronym) in use by the institution having custody of the object(s) or information referred to in the record.
collectionCode	The name, acronym, code or initialism identifying the collection or dataset from which the record was derived.
datasetName	The name identifying the dataset from which the record was derived.
basisOfRecord	The specific nature of the data record.
occurrenceID	An identifier for the Occurrence.
catalogNumber	An identifier (preferably unique) for the record within the dataset or collection.
recordedBy	A list of names of people, groups or organisations responsible for recording the original Occurrence.
associatedMedia	A list of identifiers (publication, global unique identifier, URI) of media associated with the Occurrence.
associatedReferences	A list of identifiers of literature associated with the Occurrence.
eventDate	The date-time or interval during which an Event occurred.
year	The four-digit year in which the Event occurred.
verbatimEventDate	The verbatim original representation of the date and time information for an Event.The four-digit year in which the Event occurred.
habitat	A category or description of the habitat in which the Event occurred.
continent	The name of the continent in which the Location occurs.
country	The name of the country in which the Location occurs.
countryCode	The standard code for the country in which the Location occurs.
stateProvince	The name of the next smaller administrative region than country in which the Location occurs.
county	The full, unabbreviated name of the next smaller administrative region than stateProvince.
municipality	The full, unabbreviated name of the next smaller administrative region than county in which the Location occurs.
locality	The specific description of the place.
verbatimLocality	The original textual description of the place.
verbatimElevation	The original description of the elevation (altitude) of the Location.
locationRemarks	Comments or notes about the Location.
geodeticDatum	The coordinate system and set of reference points upon which the geographic coordinates are based.
verbatimCoordinates	The verbatim original spatial coordinates of the Location.
verbatimLatitude	The verbatim original latitude of the Location.
verbatimLongitude	The verbatim original longitude of the Location.
verbatimCoordinateSystem	The spatial coordinate system for the verbatimLatitude and verbatimLongitude or the verbatimCoordinates of the Location.
decimalLatitude	The geographic latitude (in decimal degrees, using the spatial reference system given in geodeticDatum) of the geographic centre of a Location.
decimalLongitude	The geographic latitude (in decimal degrees, using the spatial reference system given in geodeticDatum) of the geographic centre of a Location.
GeoreferenceSources	A map, gazetteer or other resource used to georeference the Location.
GeoreferenceRemarks	Notes or comments about the spatial description determination, explaining assumptions made in addition or opposition to the those formalised in the method referred to in the georeferenceProtocol.
CoordinateUncertaintyInMetres	The horizontal distance (in metres) from the given decimalLatitude and decimalLongitude describing the smallest circle containing the whole of the Location.
scientificName	The full scientific name, with authorship and date information, if known.
acceptedNameUsage	The scientificName of the taxon considered to be the valid (zoological) or accepted (botanical) name for this nameUsage.
Kingdom	The full scientific name of the kingdom in which the taxon is classified.
phylum	The full scientific name of the phylum or division in which the taxon is classified.
class	The full scientific name of the class in which the taxon is classified.
order	The full scientific name of the order in which the taxon is classified.
family	The full scientific name of the family in which the taxon is classified.
genus	The full scientific name of the genus in which the taxon is classified.
specificEpithet	The name of the first or species epithet of the scientificName.
infraspecificEpithet	The name of the lowest or terminal infraspecific epithet of the scientificName, excluding any rank designation.
taxonRank	The taxonomic rank of the most specific name in the scientificName.
scientificNameAuthorship	The authorship information for the scientificName formatted according to the conventions of the applicable nomenclaturalCode.
vernacularName	A common or vernacular name.

## Supplementary Material

8B7C6840-4273-5EC4-A72D-6219AFC10839 10.3897/BDJ.9.e75951.suppl1 Supplementary material 1 Number of records (Nº R.) of each lichen species. Data type Occurrences Brief description The table shows the compilation of lichens species with the number of records in the Aysén Region. File: oo_587272.csv https://binary.pensoft.net/file/587272 Sánchez-Jardón, L.

DA990BCE-A45D-5348-BFD1-3D9BFD2E5876 10.3897/BDJ.9.e75951.suppl2 Supplementary material 2 Number of records (Nº R) of each macrofungi species. Data type Occurrences Brief description The table shows the compilation of macrofungi species with the number of records in the Aysén Region. File: oo_587277.csv https://binary.pensoft.net/file/587277 Sánchez-Jardón, L.

## Figures and Tables

**Table 1.** Number of records (Nº R.) for each Order.

**Order of Macrofungi**	**NºR**	**Order of Lichen**	**NºR**
Agaricales	203	Lecanorales	437
Pezizales	27	Peltigerales	375
Boletales	19	Pertusariales	20
Polyporales	16	Baeomycetales	14
Cyttariales	12	Teloschistales	14
Russulales	12	Rhizocarpales	13
Geastrales	7	Caliciales	12
Helotiales	7	Umbilicariales	10
Gomphales	7	Verrucariales	7
Dacrymycetales	6	Agaricales	6
Hymenochaetales	5	Gyalectales	5
Geoglossales	4	Arthoniales	4
Xylariales	4	Graphidales	3
Phallales	3	Candelariales	1
Tremellales	3	Ostropales	1
Hysterangiales	2		
Pucciniales	2		
Auriculariales	1		
Thelephorales	1		

**Table 2.** Number of records (Nº R.) identified by Family. Other Families include those with less than five records.

**Macrofungi families**	**N°R**	**Lichens families**	**N°R**
Strophariaceae	30	Parmeliaceae	277
Agaricaceae	28	Peltigeraceae	251
Cortinariaceae	28	Pannariaceae	83
Mycenaceae	18	Cladoniaceae	75
Tricholomataceae	15	Sphaerophoraceae	45
Cyttariaceae	12	Collemataceae	34
Lycoperdaceae	10	Stereocaulaceae	18
Marasmiaceae	10	Teloschistaceae	14
Polyporaceae	10	Trapeliaceae	14
Pyronemataceae	9	Coccotremataceae	13
Amanitaceae	7	Rhizocarpaceae	13
Geastraceae	7	Lecanoraceae	12
Gomphaceae	7	Physciaceae	11
Stereaceae	7	Umbilicariaceae	10
Bolbitiaceae	6	Verrucariaceae	7
Crepidotaceae	6	Hygrophoraceae	6
Dacrymycetaceae	6	Coccocarpiaceae	5
Pezizaceae	6	Phlyctidaceae	5
Schizophyllaceae	6	Other families	29
Suillaceae	6		
Entolomataceae	5		
Hygrophoraceae	5		
Morchellaceae	5		
Pluteaceae	5		
Other Families	87		
